# Food Security, Dietary Diversity, Dietary Patterns and the Double Burden of Malnutrition among School-Aged Children and Adolescents in Two Nigerian States

**DOI:** 10.3390/nu14040789

**Published:** 2022-02-14

**Authors:** Adeleye Abiodun Adeomi, Adesegun Fatusi, Kerstin Klipstein-Grobusch

**Affiliations:** 1 Department of Community Health, College of Health Sciences, Obafemi Awolowo University, Ile-Ife 220282, Osun State, Nigeria; adesegunfatusi@gmail.com; 2 Division of Epidemiology and Biostatistics, School of Public Health, Faculty of Health Sciences, University of the Witwatersrand, Parktown 2193, Johannesburg, South Africa; K.Klipstein-Grobusch@umcutrecht.nl; 3 School of Public Health, University of Medical Sciences, Ondo 220282, Ondo State, Nigeria; 4 Julius Global Health, Julius Center for Health Sciences and Primary Care, University Medical Center Utrecht, Utrecht University, 3584 CS Utrecht, The Netherlands

**Keywords:** double burden of malnutrition, under-nutrition, over-nutrition, dietary diversity, dietary patterns, household food insecurity, school-aged children, adolescents, sub-Sahara Africa

## Abstract

Background: Little evidence exists on the relationship between diet-related factors and child/adolescent malnutrition in Nigeria. This study aimed to assess the associations between household food insecurity (HFI), dietary diversity (DD), and dietary patterns (DP) with the double burden of malnutrition (DBM) among 6–19-year-olds in two Nigerian States. Methods: This community-based cross-sectional study was carried out among 1200 respondents (6–19 years in age) in the Gombe and Osun States of Nigeria. HFI was assessed using the HFI access scale. DD was assessed using a 24-h dietary recall. DP were determined by principal component analysis using a 30-day food frequency questionnaire. DP scores were categorized into quartiles (Q) for statistical analysis. Diet-related predictors of DBM were assessed using logistic regression. Results: HFI was experienced by 568 (47.3%) respondents. The median DD score was 7.0 (maximum of 14). Two DPs were identified, diversified DP (DDP) and traditional DP (TDP). TDP was significantly associated with both thinness (Q4:OR: 2.91; 95% CI: 1.52–5.55; Ptrend: 0.002) and overweight/obesity (Q4:OR: 2.50; 95% CI: 1.43–4.35; Ptrend: 0.007), while DDP was inversely related with thinness (Q4:OR: 0.36; 95% CI: 0.21–0.61; Ptrend: 0.008) as compared to Q1. Conclusions: TDP increased the odds for DBM, while the DDP reduced the odds.

## 1. Introduction

There has been a steady rise in the prevalence of overweight and obesity among children in all regions of the world. Prevalence rates in the range of 10–40% for overweight/obesity were reported in selected high-income countries among 15-year-old adolescents [1]. The World Health Organization (WHO) reports that 340 million school-aged children and adolescents (aged 5–19 years) were overweight/obese globally in 2016 [2]. Low- and middle-income countries have also experienced significant rise in the prevalence of overweight/obesity with highest prevalence rates recorded in the Middle East, North Africa, Latin America and the Caribbean [1,3].

Despite the rising prevalence of overweight/obesity in sub-Saharan Africa, the prevalence of underweight children still remains high. In a study that assessed the worldwide trend in the nutritional status of 24.1 million children between the ages of 5–17 years from 1975 to 2016, the prevalence of underweight was observed to have been reduced in virtually all regions, except for South Asia, Central Africa, East Africa, and West Africa [2]. Thus, most countries in sub-Saharan Africa, including Nigeria, are experiencing what has been termed as “double burden of malnutrition”, which is the co-existence of both under- and over-nutrition [4,5].

Identifying the determinants of under- and over-nutrition is important not only in improving the understanding about the subject, but also critical for planning appropriate nutritional interventions for affected children. However, to date, most of the research efforts targeted at identifying the determinants of the nutritional status of school-aged children and adolescents in Nigeria have focused on socio-demographic factors [6,7,8,9]. Little evidence could be found in the literature on the relationship between the nutritional status of school-aged children and adolescents and key factors such as food security, dietary diversity and dietary patterns in Nigeria. Thus, a significant evidence gap exists with regards to determinants of nutritional status of school children and adolescents in Nigeria, particularly as diet is reported as an important factor in the epidemiology of childhood and adolescent malnutrition [10,11,12].

Household food security, dietary diversity and dietary patterns are methods that are used to describe the diet and food consumption at individual and household levels. Household food security refers to situations when a household has access to sufficient quantity and quality of food for all members of the household for a given period [13]. Dietary diversity qualitatively measures access and consumption of varieties of food types as a proxy for nutrient/diet adequacy and quality at individual and household levels [14]. Dietary patterns, on the other hand, describe a broader and more comprehensive conceptualization of food consumption as against the traditional method of finding the frequency of the consumption of individual food types or nutrients [15]. Each of these three elements are shown to be associated with nutritional status and studies have also shown relationships between them [16,17].

Few studies have assessed household food insecurity in Nigeria [18,19], and fewer still have directly linked household food insecurity to the nutritional status of school-aged children or adolescents. Likewise, dietary diversity and its relation to under- and over-nutrition among school-aged children or adolescents has only been assessed in few Nigerian studies [20,21,22]. Data on the dietary patterns of school-aged children and adolescents in Nigeria is even more scarce. Previous reports from Nigeria who attempted to describe the dietary patterns of children and/or adolescents only described the frequency of consumption of different food groups or the dietary habits of the children or adolescents [23,24,25]. Only one recently published article described the dietary patterns of a small sample of out-of-school adolescents [26] in Nigeria using dietary pattern analysis [15].

The present study aims to address the evidence gap highlighted above: it aimed to assess household food security, dietary diversity and dietary patterns in relation to under- and over-nutrition among school-aged children and adolescents in two selected states in Nigeria located in two different parts of the country (North and South). 

## 2. Materials and Methods

### 2.1. Study Location

The study was carried out in two randomly selected states in Nigeria. Nigeria has 36 states and is a multi-ethnic nation with significant socio-economic and cultural differences between its two broad geographical areas: the north and the south. The dominant ethnic groups in the north are the Hausas and Fulanis, while the dominant groups in the southern part of Nigeria are Yorubas and Igbos. Nigeria is politically structured into 6 geo-political zones that are equally distributed between the northern and southern parts of the country. For this study, the geo-political zones with the lowest (North-East) and highest (South-West) wealth index based on the 2018 Nigeria Demographic and Health Survey (NDHS) [27] were selected, and Gombe and Osun States were randomly selected from the two zones, respectively. 

### 2.2. Study Design, Population and Size

A community-based cross-sectional study was carried out among 1200 school-aged children and adolescents aged 6–19 years (600 from each of the selected states). School-aged children are usually defined as those 6 to 11 years of age [28], or 5 to 14 years [29], while adolescents are those from 10–19 years of age [29]. In the present study, school-aged children and adolescents are taken as those from 6 to 19 years of age. The sample size was determined using STATCALC on the Epi-Info software, [30] to get an absolute precision of ±5%. The proportion of expected outcome was taken as 33%, which is the prevalence of stunted children in Nigeria [31], and a design effect of 1.5 was used because of the hierarchical sampling model. The calculated sample size for each of the states was 510, and after correcting for an anticipated 10% non-response, the same sample became 561, which was rounded off to 600 for each of the states (making a total of 1200). The sample size determination was previously described in detail in another study [32].

### 2.3. Sampling Technique

Respondents were selected using multi-stage sampling technique. At the first stage, two local government areas (LGAs) were selected from each of the two selected states (one from rural and one from urban LGAs) using a simple random sampling technique (Balloting method). Two wards/districts each were randomly selected from each LGA at the second stage. In each of the selected wards/districts, five enumerations areas (EAs), as demarcated by the National Population Commission for the 2006 population census, were randomly selected for the third stage. At the fourth stage, the listing of the households in the EAs was first carried out, then 30 households were drawn in each of the selected EAs using systematic sampling technique. At the household level, one school-age child or adolescent was selected. If a household had more than one school-aged child or adolescent who met the inclusion criteria, only one was randomly selected.

### 2.4. Data Collection

Ten research assistants (five males and five females) and one field supervisor were recruited and trained to collect data in each of the two states. The questionnaires were administered with RedCap [33] and the anthropometric measurements, i.e., the weight and height of the children/adolescents were taken using standard protocols recommended by the International Society for the Advancement of Kinanthropometry [34].

### 2.5. Outcome Variable

The primary outcome/dependent variable is the nutritional status, which was assessed with the WHO 2007 reference values, [35] using the BMI-for-age Z-scores and categorized into: (1) thinness, (2) normal, and (3) overweight/obese for BMI-for-age Z-scores <−2, −2 to 1, >1 respectively. This was re-categorized into two different outcome variables for under-nutrition (i.e., (1) thinness, (0) otherwise) and over-nutrition (i.e., (1) overweight/obese, (0) otherwise). 

### 2.6. Explanatory Variables

#### 2.6.1. Household Food Insecurity

Household food security was measured using the household food insecurity access scale (HFIAS) [36]. The HFIAS has nine occurrence questions with a recall period of four weeks (30 days), and these are followed by three frequency-of-occurrence questions to determine whether the condition happened rarely (once or twice), sometimes (three to ten times) or often (more than ten times) in the past four weeks. A response of “No” to the frequency-of-occurrence questions was scored 0, while “rarely”, “sometimes”, and “often” were scored 1, 2, and 3 respectively. The households were then categorized into “food secure”, “mildly food insecure”, “moderately food insecure” and “severely food insecure”, using the responses to these questions and based on extant literature [36]. These four categories were re-categorized into “food secure” (food secure and mildly food insecure) and “food insecure” (moderately and severely food insecure) households for data analysis. 

#### 2.6.2. Dietary Diversity (DD)

A scale of 14 food groups including the following was used in assessing the DD score for each of the children and adolescents; [14,21] cereals, vitamin A vegetables and tubers, white tubers, dark green leafy vegetables, other vegetables, vitamin A fruits, other fruits, organ meat, flesh meat, egg and egg products, fish, legumes/nuts/seeds, milk/milk products and oils/fats. DD classification was based on a one-time 24-h dietary recall. This involved the respondents listing all the foods and drinks they had taken in the 24 h preceding the data collection. Using the information collected from the 24-h dietary recall, a point was awarded to each food group consumed out of the 14 food groups, and the sum of all the points awarded was the DD score for each child. Hence the DD score ranged from a minimum of 0 (if no food from the 14 food groups was consumed) and 14 (if food from all 14 food groups was consumed). The DD scores lower than the median score of seven were grouped as “low”, while others (≥7) were regarded as high. 

#### 2.6.3. Dietary Patterns (DP)

**Figure 1:**
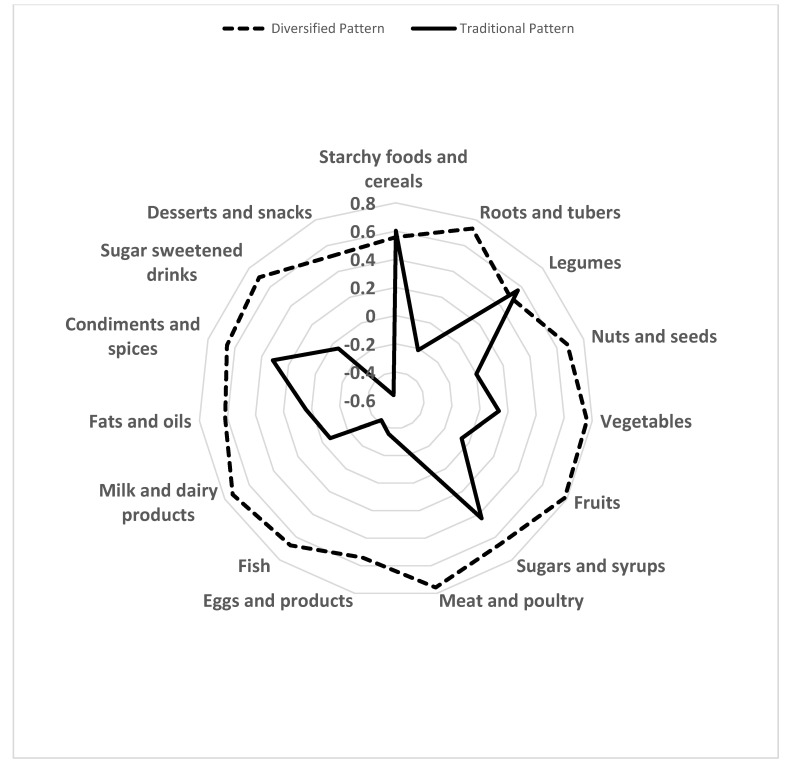
Radar chart showing the two dietary patterns, the 15 food groups and their factor loadings among school-aged children and adolescents in two States in Nigeria.

A 30-day food frequency questionnaire (FFQ) was used to collect information on the frequency and amount of 92 different food items consumed in the last 30 days preceding the study. The FFQ was adapted from the one used among school-aged children in Ghana [37] and further modified after pre-testing the research instrument. For the present study, the 92 different food items were collapsed into 15 food groups, based on their nutritional profile (Supplementary Table S1). Principal component analysis (PCA) with Varimax rotation was used to determine the dietary patterns which best represented the food intake of the population. The number of patterns was determined using the Scree plot and eigenvalues values greater than 1.0. Two components (i.e., patterns) explained 56% of the total variance and were retained. Food groups with absolute loadings greater or equal to 0.4 were used to name the retained principal components/patterns (Figure 1). The first pattern showed positive loadings greater than 0.4 for all food groups and hence was described as “*Diversified dietary pattern*”. The second pattern showed positive loadings greater than 0.4 for starchy foods/cereals, legumes and sugars and negative loadings for fish, desserts and snacks, and was described as the “*Traditional dietary pattern*”, because the typical traditional diet in Nigeria is dominated by cereals/starchy foods and legumes. The pattern scores generated for each respondent by PCA were categorized into quartiles for the statistical analysis. 

#### 2.6.4. Other Explanatory Variables

Socio-demographic characteristics, which include age, sex, household wealth index, residence, state and ethnicity, were included in the variables for the analysis. The household wealth index was calculated using household possessions through PCA and categorized into tertiles, (1) high, (2) middle, and (3) low, as earlier explained in another article. [32] Pubertal staging was assessed using the Tanner pubertal self-rating scale [38]. The scale has a score range of 1–5, and the respondents were grouped into early puberty (Tanner stage 2 and below) and mid-puberty (>Tanner stage 2). Physical activity was assessed using the physical activity questionnaire for older children and adolescents by Kowalski et al. [39] which was measured as scores from 1 to 5. The higher the score, the more active the respondent was, with 1 representing the option with least or no activity and 5 being the option with most activity. 

### 2.7. Data Analysis

Data from the RedCap database were exported and analyzed using Stata version 15. All the dependent and independent variables were initially described, after which Pearson chi-square was used to test for associations for categorical variables at bivariate level. The Kruskal–Wallis test was used to test the associations between nutritional status and continuous variables at bivariate level, because the variables were not normally distributed. Four models each were fitted for food insecurity, dietary diversity, diversified food pattern and the traditional food pattern, and their relationships with thinness and overweight/obesity using binary logistic regression analysis. Model 0 was the empty model showing crude/unadjusted rates, while Model 1 adjusted for age and sex. Model 2 adjusted for State of residence and household wealth index in addition to Model 1, and Model 3 was the full model that adjusted for physical activity scores in addition to Model 2. Variables with high variance inflation factor (VIF) when multi-collinearity diagnostics were performed were not included in the models. Therefore, pubertal staging, residence and ethnicity were not included to prevent redundancy. The level of significance was set at *p* < 0.05.

### 2.8. Ethical Considerations

The study was conducted according to the guidelines of the Declaration of Helsinki and approved by the Human Research Ethics Committee of the University of the Witwatersrand, South Africa (certificate No: M190514, approved on 25/09/2019), and the Ministry of Health in Osun State (certificate No: OSHREC/PRS/569T/155, approved on 24/06/2019) and Gombe State (certificate No: MOH/ADM/621/1/142, approved on 26/07/2019) in Nigeria. Informed consent was obtained from all subjects involved in the study. Written consent was obtained from adolescents who were 18 years and above, and the parents of children less than 18 years, while assent was obtained in addition from children less than 18 years. All severely malnourished children were referred to the nearby public health facilities for further management.

## 3. Results

The distribution of the dependent and independent variables by the state of residence is shown in Table 1. The prevalence rate of thinness was 10.3% and overweight/obesity was 11.4%. At bivariate level, thinness (under-nutrition) had statistically significant associations with ethnicity (*p* < 0.001), household wealth index (*p* < 0.001), state of residence (*p* < 0.001), residence (rural/urban) (*p* = 0.0013), diversified dietary pattern (*p* < 0.001) and traditional dietary pattern (*p* = 0.0013). Overweight/obesity had significant statistical relationships with age (*p* < 0.001), gender (*p* < 0.001), ethnicity (*p* < 0.001), state of residence (*p* < 0.001), residence (rural/urban) (*p* < 0.001) and physical activity (*p* = 0.001).

Figure 1 shows the diversified and traditional dietary patterns, and their rotated factor loadings using a radar chart. The diversified dietary pattern was characterized by a high intake (i.e., factor loading ≥ 0.4) of all listed food groups, while the traditional pattern had a high intake of starchy foods/cereals, legumes and sugars, and very low intake (i.e., factor loading ≤ 0.4) of fish, desserts and snacks. The diversified dietary pattern explained 44.1% of the variance, while the traditional dietary pattern explained 11.5% of the variance. 

The socio-demographic characteristics, physical activity, food insecurity and dietary diversity across the quartiles of the two dietary patterns is presented in Table 2. All the considered variables, except age (*p* = 0.926) and sex (*p* = 0.571), had statistically significant association with the diversified dietary pattern (*p* ≤ 0.001). The traditional dietary pattern had a statistically significant association with all the considered variables (*p* < 0.05) except pubertal staging (*p* = 0.637) and food insecurity (0.403).

Table 3 shows adjusted odds ratios and 95% CIs from the associations of food insecurity and dietary diversity, with thinness (under-nutrition) and overweight/obesity (over-nutrition) among the respondents. There were no statistically significant associations across all the models for both food insecurity and dietary diversity.

The adjusted odds ratios and 95% CIs from the associations of the diversified and traditional dietary patterns with thinness (under-nutrition) and overweight/obesity (over-nutrition) among the respondents is shown in Table 4. With respondents in the first quartile being the reference, respondents in all other quartiles had a statistically significant association with under-nutrition (thinness) at the crude/empty model level; the diversified dietary pattern was inversely associated (p_trend_ = 0.009) while the traditional dietary pattern was positively associated (p_trend_ = 0.002). However, only those in the second quartile of the diversified dietary pattern retained the statistically significant association after controlling for all the independent variables (i.e., in the full model/Model 3) (OR: 0.44; *p* = 0.007; 95% CI: 0.24 to 0.80). For the association between dietary patterns and over-nutrition (overweight/obesity), there was no significant associations at the crude model level, but the full models of Quartile 2 (OR: 2.06; *p* = 0.009; 95% CI: 1.20 to 3.55) and 4 (OR: 2.50; *p* = 0.001; 95% CI: 1.43 to 4.35) of the traditional dietary pattern showed statistically significant positive association. The P_trend_ was 0.007, which indicates a statistically significant linear association.

## 4. Discussion

Few scattered small-scale studies exist regarding diet-related factors among school-aged children and adolescents in Nigeria, but no study was found that considered these factors together and related them to under-and over-nutrition among older children. Addressing these factors together is important so as to assess and control for any potential confounding effects in explaining the associations between the explanatory and outcome variables. Existing studies also have methodological limitations of unrepresentative study populations and/or unconventional methodologies that make comparisons of the results difficult. The current study has the advantage of avoiding these limitations: in particular, it was based on representative populations from two states of Nigeria that are diverse geographically, socio-culturally, and economically, and used well established approaches, with rigorous analysis in addressing the association of diet related factors with under- and over-nutrition. 

Nearly half of the households were moderately or severely food insecure. Previous studies on household security in Nigeria have similarly reported rates of moderate/severe food insecurity in excess of 50% [18,40]. These findings are disturbing, especially as this was found to be significantly associated with the nutritional status of the children or adolescents living in such households. [40] The median DD score for the present study was 7 out of a total possible score of 14, with two-thirds of the respondents scoring 7 or below. Other previous studies on dietary patterns among children or adolescents reported similar findings with the mean/median DD score being about half of the total DD score [20,22,41]. The present study found that half of the respondents had dietary diversity scores less than 7, which is just half of the maximum value of 14. This indicates sub-optimum dietary diversity among half of the respondents. 

Two dietary patterns were identified in the present study, which were named diversified and traditional DP. The only other study on dietary pattern among adolescents in Nigeria using the principal component analysis/factor analysis had very similar findings. The authors reported two patterns also, with one characterized by a high intake of all the food groups that were considered while the other was characterized by a high intake of roots/tubers and legumes, and they called these the healthy and unhealthy DPs, respectively [26]. A study from Ghana also reported two DPs, but with very different food group loadings from the present study and the previous Nigerian study [26]. The DPs described for adolescents from other countries [42,43] outside Africa were also quite different from those found among school-aged children or adolescents in Nigeria and Ghana [26,44]. 

The diversified dietary pattern had an inversely significant association with thinness, even after adjusting for all independent variables, i.e, showing 56% lower odds of thinness in those in the fourth as compared to those in the first quartile. The inverse relationship between the diversified dietary pattern and thinness is expected because the diversified diet reflects a healthier dietary pattern. Even though not statistically significant, it may be important to also note that that the diversified dietary pattern was also inversely related to overweight/obesity. This underscores the importance of a healthy or diversified dietary pattern for better health (i.e., lower odds for thinness and overweight/obesity). 

The traditional dietary pattern had a positively significant association with overweight/obesity, such that those in the second and fourth quartiles of the traditional dietary pattern had 2 times higher odds of being overweight/obese than those in the first quartile. The traditional dietary pattern also had a positive association with thinness, although the relationship was only statistically significant in the crude model. Overall, the traditional dietary pattern seems to increase the odds of thinness and overweight/obesity, therefore, contributing to the double burden of malnutrition. This may not be unexpected because the traditional dietary pattern is characterized by higher intake of starchy foods/cereals and sugars. This finding further underscores the importance of a healthy dietary pattern in combating the double burden of malnutrition among school-aged children and adolescents in these two states, and probably the whole of Nigeria. 

Similarly, different studies carried out in other countries have reported associations between dietary patterns and under- or over-nutrition [42,43,45], though the pattern of association is inconsistent. The most diversified DP in a study among Spanish adolescents had lower odds for overweight/obesity [43], while a study carried out in Bangladesh found that the diversified DPs were significantly and positively associated with overweight/obesity [42]. Alangea et al. [44] in Ghana found a significant association between the energy-dense DP and childhood overweight/obesity. Meanwhile, studies by Abizari et al. [46] and Samuel et al. [26] in Ghana and Nigeria, respectively, found no association between the dietary patterns and nutritional status of the adolescents. This lack of consensus on the nature of the relationship between dietary patterns and body mass index was previously noted [43]. This may not be un-expected because of the multi-factorial and complex nature of the determinants of nutritional status. The role cultural, ethnic and religious factors [47,48,49,50], and influence of contextual factors such as household level and community level factors [51,52] cannot be ruled out. Furthermore, the role of indirect associations can also not be ruled out, in the present study as an example, the indirect association with food insecurity and dietary diversity are also important. More research is thus needed to understand the mechanism underlying the associations. 

The relationships between food insecurity, dietary diversity and thinness and overweight/obesity were not statistically significant. However, indirect associations cannot be ruled out because of the significant associations that existed between the dietary patterns and both food insecurity and dietary diversity at the bivariate analysis level. School-aged children and adolescents who came from food insecure homes, and those who had low dietary diversity were significantly less likely to have a diversified dietary pattern, while a third of those in the fourth quartile, and more than half of those in the third and fourth quartiles of the traditional dietary pattern were food insecure. This shows a possible relationship between food insecurity and the unhealthy (i.e., traditional) dietary pattern which had a positive association with the double burden of malnutrition. 

Thinness and overweight/obesity among the school-aged children and adolescents in this present study were significantly associated with variables that were not diet-related. The variables who had significant associations with both dietary patterns in this study were ethnicity, household wealth index, state and residence. These relationships underscore the importance of the social determinants of health. Social determinants have a major influence on health and health related behavior, and especially on diet and/or nutrition of people [53,54,55]. Religion, for instance, dictates some food that are to be eaten, and others that should be avoided, and this is irrespective of other variables including the wealth index of such households. Similarly, previous researchers have found that ethnicity, culture and other social determinants influence dietary patterns [47,48]. In the present study, those in the high household wealth index category, for example, were more likely to have a diversified diet, and this may indicate that household financial means determines affordability of food and hence determines dietary patterns. 

While the present study involved a representative sample from two states, the findings may not be generalizable to the entire country of 36 states, particularly granted the social diversity, cultural plurality, and multi-ethnic nature of Nigeria. Additionally, the assessment of dietary diversity and dietary patterns was dependent on the recall of food taken in the last 24 h and the last 30 days respectively, which makes them prone to recall bias [56,57]. The aetiology of under- and over-nutrition is complex, and this study could not assess all possible explanatory variables, hence the possibility of residual confounding could not be ruled out. 

## 5. Conclusions

A traditional dietary pattern (containing mainly cereals/starchy food and legumes) significantly increased the odds for both thinness and overweight/obesity, while a diversified dietary pattern (containing all food groups) significantly reduced the odds for thinness in school-aged children and adolescents in Nigeria. Dietary patterns were the only diet-related factors that had direct associations with under- and over-nutrition, while food insecurity and dietary diversity had indirect associations. DBM was also associated with socio-demographic and socio-economic variables, underscoring the importance of the social determinants of health. Nutrition education programmes to promote a healthy diversified dietary pattern that will reduce the burden of under- and over-nutrition among age 6–19 years needs to be intensified in Nigeria for school-aged children, adolescents and their parents. 

## Figures and Tables

**Table 1:** Description of the study population in Gombe and Osun States (*n* = 1200).

Variables	States		
	^f^ Gombe *n* (%)	^f^ Osun *n* (%)	Total *n* (%)
Age of the child (IR)	12.0 (7.0)	11.0 (5.0)	11.0 (6.0)
^a^ BMI-for-age			
Thinness	83 (13.8)	40 (6.7)	123 (10.3)
Normal	476 (79.3)	464 (77.3)	940 (78.3)
Overweight/Obesity	41 (6.8)	96 (16.0)	137 (11.4)
Sex			
Male	323 (53.8)	278 (46.3)	601 (50.1)
Female	277 (46.2)	322 (53.7)	599 (49.9)
Pubertal staging			
Early puberty	379 (63.2)	355 (59.2)	734 (61.2)
Mid puberty	221 (36.8)	245 (40.8)	466 (38.8)
Ethnicity			
Yoruba	65 (10.8)	574 (95.7)	639 (53.3)
Igbo	23 (3.8)	15 (2.5)	38 (3.2)
Hausa	150 (25.0)	0 (0.0)	150 (12.5)
Fulani	144 (24.0)	3 (0.5)	147 (12.3)
Minorities	218 (36.3)	8 (1.3)	226 (18.8)
^b^ Household wealth index			
Low	205 (34.2)	195 (32.5)	400 (33.3)
Middle	189 (31.5)	211 (35.2)	400 (33.3)
High	206 (34.3)	194 (32.3)	400 (33.3)
Residence			
Rural	300 (50.0)	300 (50.0)	600 (50.0)
Urban	300 (50.0)	300 (50.0)	600 (50.0)
^c^ Food security			
Food secure	320 (53.3)	312 (52.0)	632 (52.7)
Food insecure	280 (46.7)	288 (48.0)	568 (47.3)
^d^ Dietary diversity			
Low	244 (41.1)	341 (57.0)	585 (49.1)
High	350 (58.9)	257 (43.0)	607 (50.9)
^e^ Diversified dietary pattern			
Quartile 1	249 (41.5)	52 (8.7)	301 (25.1)
Quartile 2	150 (25.0)	149 (24.8)	299 (24.9)
Quartile 3	112 (18.7)	188 (31.3)	300 (25.0)
Quartile 4	89 (14.8)	211 (35.2)	300 (25.0)
^e^ Traditional dietary pattern			
Quartile 1	32 (5.3)	268 (44.7)	300 (25.0)
Quartile 2	165 (27.5)	135 (22.5)	300 (25.0)
Quartile 3	195 (32.5)	105 (17.5)	300 (25.0)
Quartile 4	208 (34.7)	92 (15.3)	300 (25.0)

IR—interquartile range; BMI—body mass index. ^a^ Categorized using BMI-for-age Z-scores; thinness (<−2), normal (−2 to 1) and overweight/obesity (>1) ^b^ Household wealth index scores were derived from scoring the possession of household commodities using principal component analysis, which were then categorized into three (low/middle/high) ^c^ Measured using HFIAS, “food secure” represents those that were food secure and mildly food insecure, while “food insecure” represents moderately and severely food insecure) ^d^ Those with a dietary diversity score lower than the median score of 7 were grouped as “low”, while others (≥7) were regarded as high. ^e^ Dietary pattern scores derived using principal component analysis were categorized into quartiles. ^f^ The number of respondents in each of Gombe and Osun States is 600.

**Table 2:** Demographic variables, physical activity, food insecurity and dietary diversity across the quartiles of the two dietary patterns.

Variables	Diversified Dietary Pattern				*p*-Value	Traditional Dietary Pattern				*p*-Value
	Q1	Q2	Q3	Q4		Q1	Q2	Q3	Q4	
^a^ Age	**11.0 (6.0)**	11.0 (5.0)	12.0 (7.0)	12 (6.0)	0.926	10.0 (5.0)	11.0 (6.0)	11.5 (6.0)	12.0 (6.0)	<0.001 *
^a^ Physical Activity Scores	2.1 (1.0)	2.0 (1.0)	2.4 (1.1)	2.6 (0.9)	<0.001 *	2.2 (1.2)	2.4 (1.0)	2.2 (1.1)	2.3 (0.9)	0.034 *
Sex					0.571					<0.001 *
Male	152 (25.3)	159 (26.5)	143 (23.8)	147 (24.5)		121 (20.1)	158 (26.3)	151 (25.1)	171 (28.5)	
Female	149 (24.9)	140 (23.4)	157 (26.2)	153 (25.5)		179 (29.9)	142 (23.7)	149 (24.9)	129 (21.5)	
Pubertal Staging					0.001 *					0.637
Early Puberty	211 (28.7)	184 (25.1)	168 (22.9)	171 (23.3)		187 (25.5)	187 (25.5)	186 (25.3)	174 (23.7)	
Mid Puberty	90 (19.3)	115 (24.7)	132 (28.3)	129 (27.7)		113 (24.2)	113 (24.2)	114 (24.5)	126 (27.0)	
Ethnicity					<0.001 *					<0.001 *
Yoruba	65 (10.2)	159 (24.9)	203 (31.8)	212 (33.2)		264 (41.3)	139 (21.8)	125 (19.6)	111 (17.4)	
Igbo	3 (7.9)	11 (28.9)	9 (23.7)	15 (39.5)		7 (18.4)	18 (47.4)	2 (5.3)	11 (28.9)	
Hausa	62 (41.3)	35 (23.3)	28 (18.7)	25 (16.7)		12 (8.0)	51 (34.0)	37 (24.7)	50 (33.3)	
Fulani	83 (56.5)	25 (17.0)	23 (1.6)	16 (10.9)		7 (4.8)	42 (28.6)	48 (32.7)	50 (34.0)	
Minorities	88 (38.9)	69 (30.5)	37 (16.4)	32 (14.2)		10 (4.4)	50 (22.1)	88 (38.9)	78 (34.5)	
Household Wealth Index					<0.001 *					<0.001 *
Low	170 (42.5)	68 (17.0)	75 (18.8)	87 (21.8)		75 (18.8)	125 (31.3)	94 (23.5)	106 (26.5)	
Middle	88 (22.0)	115 (28.7)	116 (29.0)	81 (20.3)		116 (29.0)	86 (21.5)	122 (30.5)	76 (19.0)	
High	43 (10.8)	116 (29.0)	109 (27.3)	132 (33.0)		109 (27.3)	89 (22.3)	84 (21.0)	118 (29.5)	
State					<0.001 *					<0.001 *
Gombe	249 (41.5)	150 (25.0)	112 (18.7)	89 (14.8)		32 (5.3)	165 (27.5)	195 (32.5)	208 (34.7)	
Osun	52 (8.7)	149 (24.8)	188 (31.3)	211 (35.2)		268 (44.7)	135 (22.5)	105 (17.5)	92 (15.3)	
Residence					<0.001 *					<0.001 *
Rural	109 (18.2)	123 (20.5)	181 (30.2)	187 (31.2)		174 (29.0)	135 (22.5)	129 (21.5)	162 (27.0)	
Urban	192 (32.0)	176 (29.3)	119 (19.8)	113 (18.8)		126 (21.0)	165 (27.5)	171 (28.5)	138 (23.0)	
Food Security					<0.001 *					0.403
Food Secure	291 (26.3)	259 (23.4)	271 (24.5)	286 (25.8)		280 (25.3)	278 (25.1)	279 (25.2)	270 (24.4)	
Food Insecure	10 (10.8)	40 (43.0)	29 (31.2)	14 (15.1)		20 (21.5)	22 (23.7)	21 (22.6)	30 (32.3)	
Dietary Diversity					<0.001 *					<0.001 *
Low	198 (33.8)	153 (26.2)	130 (22.2)	104 (17.8)		175 (29.9)	171 (29.2)	150 (15.6)	89 (15.2)	
High	97 (16.0)	146 (24.1)	168 (27.7)	196 (32.3)		125 (20.6)	127 (20.9)	144 (23.7)	211 (34.8)	
^a^ Dietary Diversity Score	5.0 (4.0)	6.0 (3.0)	7.0 (4.0)	8.0 (7.0)	<0.001 *	6.0 (3.0)	6.0 (4.0)	6.0 (5.0)	9.0 (6.0)	<0.001 *

Q—quartiles; *—statistically significant; ^a^—Kruskal–Wallis test was used because the variables were not normally distributed, and median (inter-quartile range) was used to describe the data.

**Table 3:** The associations of food insecurity and dietary diversity, with thinness (under-nutrition) and overweight/obesity (over-nutrition) among school-aged children and adolescents in two Nigerian states, using binary logistic regression (*n* = 1200).

^a^ Models	^b^ Ref	OR	95% CI	*p*-Value
Food Insecurity				
Model 0 (Empty/Crude)	1	1.33	0.71, 2.51	0.381
Model 1	1	1.35	0.72, 2.57	0.351
Model 2	1	1.34	0.70, 2.58	0.377
Model 3	1	1.34	0.70, 2.57	0.382
Dietary Diversity				
Model 0 (Empty/Crude)	1	0.85	0.58, 1.23	0.378
Model 1	1	0.83	0.57, 1.21	0.324
Model 2	1	0.91	0.61, 1.36	0.645
Model 3	1	0.91	0.61, 1.35	0.637
Food Insecurity				
Model 0 (Empty/Crude)	1	0.71	0.34, 1.51	0.376
Model 1	1	0.7	0.33, 1.50	0.358
Model 2	1	0.79	0.37, 1.70	0.546
Model 3	1	0.73	0.33, 1.60	0.433
Dietary iversity				
Model 0 (Empty/Crude)	1	1.04	0.73, 1.49	0.822
Model 1	1	1.23	0.85, 1.77	0.275
Model 2	1	1.31	0.90, 1.92	0.158
Model 3	1	1.34	0.91, 1.96	0.134

OR–odds ratio; CI–confidence interval; ^a^ Model 1–adjusted for age and sex; Model 2: Model 1 + household wealth index and state; Model 3: full model (i.e., Model 2 + physical activity scores); ^b^ represents the food secure category and the category with low dietary diversity which served as reference values for food insecurity and dietary diversity respectively.

**Table 4:** The associations of the diversified and traditional dietary patterns, with thinness (under-nutrition) and overweight/obesity (over-nutrition) among school-aged children and adolescents in two Nigerian states, using binary logistic regression (*n* = 1200).

^a^ Models	Q1 (Ref)	Q2			Q3			Q4			*p*-Trend
		OR	*p*-Value	95% CI	OR	*p*-Value	95% CI	OR	*p*-Value	95% CI	
**Thinness**											
Diversified Dietary Pattern											
Model 0 (Empty/Crude)	1	0.28	<0.001 *	0.16, 0.49	0.51	0.006 *	0.31, 0.82	0.36	<0.001 *	0.21, 0.61	0.009 *
Model 1	1	0.27	<0.001 *	0.15, 0.48	0.51	0.006 *	0.32, 0.82	0.36	<0.001 *	0.21, 0.61	0.008 *
Model 2	1	0.44	0.007 *	0.24, 0.80	0.89	0.675	0.52, 1.53	0.72	0.285	0.40, 1.31	0.915
Model 3	1	0.44	0.007 *	0.24, 0.80	0.91	0.737	0.53, 1.57	0.75	0.343	0.41, 1.37	0.827
Traditional Dietary Pattern											
Model 0 (Empty/Crude)	1	2.96	0.001 *	1.57, 5.59	2.61	0.004 *	1.37, 4.97	2.87	0.001 *	1.52, 5.44	0.002 *
Model 1	1	2.98	0.001 *	1.57, 5.63	2.63	0003 *	1.38, 5.04	2.91	0.001 *	1.52,5.55	0.002 *
Model 2	1	1.94	0.059	0.97, 3.86	1.63	0.177	0.80, 3.32	1.95	0.065	0.96, 3.96	0.114
Model 3	1	1.99	0.051	1.00, 3.98	1.64	0.171	0.81, 3.98	1.98	0.059	0.97, 4.03	0.106
**Overweight/Obesity**											
Diversified Dietary Pattern											
Model 0 (Empty/Crude)	1	1.01	0.978	0.60, 1.70	1.42	0.166	0.86, 2.32	1.08	0.781	0.64, 1.81	0.871
Model 1	1	1.01	0.957	0.60, 1.73	1.39	0.193	0.84, 2.30	1.09	0.742	0.65, 1.85	0.935
Model 2	1	0.66	0.164	0.36, 1.19	0.83	0.529	0.47, 1.48	0.6	0.1	0.33, 1.10	0.1
Model 3	1	0.62	0.119	0.34, 1.13	0.92	0.78	0.51, 1.65	0.78	0.421	0.42, 1.44	0.581
Traditional Dietary Pattern											
Model 0 (Empty/Crude)	1	0.97	0.901	0.59, 1.58	0.7	0.188	0.42, 1.19	1	1	0.61, 1.63	0.865
Model 1	1	1.14	0.606	0.69, 1.88	0.83	0.488	0.49, 1.41	1.31	0.29	0.79, 2.17	0.444
Model 2	1	1.7	0.048 *	1.01, 2.87	1.33	0.316	0.76, 2.34	2.14	0.006 *	1.24, 3.67	0.017 *
Model 3	1	2.06	0.009 *	1.20, 3.55	1.5	0.169	0.84, 2.66	2.5	0.001 *	1.43, 4.35	0.007 *

Ref—reference value; OR—odds ratio; CI—confidence interval; *—statistically significant Q—represent quartiles, which were derived from the principal component analysis scores for both the diversified and traditional dietary patterns ^a^ Model 1—adjusted for age and sex; Model 2—Model 1 + household wealth index and state; Model 3: full model (i.e., Model 2 + physical activity scores).

## Data Availability

The data presented in this study are available on request from the corresponding author. The data are not publicly available due to ethical considerations.

## Supplementary Materials

The following are available online at https://www.mdpi.com/article/10.3390/nu14040789/s1. Table S1: List of food types and their groups used for the assessment of dietary patterns.
