# Supplementary material for: Food Security, Dietary Diversity, Dietary Patterns and the Double Burden of Malnutrition among School-Aged Children and Adolescents in Two Nigerian States
Source: Nutrients. 2022 Feb 14;14(4):789. doi: 10.3390/nu14040789 (PMC8875779; doi:10.3390/nu14040789)
Supplement: Supplementary file 1 [file nutrients-14-00789-s001.zip › nutrients-1432767-supplementary.pdf]

Table S1: List of food types and their groups used for the assessment of dietary patterns

SN	Food types	Food groups
1.	White rice	Cereals and Grain products
2.	Brown/local rice	
3.	Maize	
4.	Millet	
5.	Sorghum/Guinea corn	
6.	Bread	
7.	Spaghetti/macaroni	
8.	Wheat	
9.	Cassava	Starchy fruits, roots and tubers
10.	Yam	
11.	Potato	
12.	Cocoyam	
13.	Plantain	
14.	Beans	Grain legumes & products
15.	Soybean	
16.	Groundnut	Nuts & seeds
17.	Cashew nuts	
18.	Coconut	
19.	Cabbage	Vegetables and products
20.	Carrot	
21.	Cucumber	
22.	Waterleaf	
23.	Mushroom	
24.	Tomato	
25.	Amaranthus leaves	
26.	Pumpkin leaves	
27.	Bitter leaves	
28.	Scent leaves	
29.	Okra	
30.	Jutw mallow	
31.	Eggplant	
32.	Moringa leaves	
33.	Spinach	
34.	Other vegetables (specify)	
35.	Apple	Fruits
36.	Avocado	
37.	Citrus fruits (e.g. orange, tangerine)	
38.	Guava	
39.	Mango	
40.	Pawpaw	
41.	Pineapple	
42.	Watermelon	
43.	African star apple	
44.	banana	
45.	Other fruits (specify)	
46.	Honey	Sugars and syrups
47.	Sugar	

48.	Sugar cane	
49.	Beef (cow meat)	Meat & poultry
50.	organ meat	
51.	Cow skin	
52.	Chicken	
53.	Turkey	
54.	Goat	
55.	Guinea fowl	
56.	Pork	
57.	Sheep/lamb	
58.	Rabbit	
59.	Snail	
60.	Duck	
61.	Bush meat	
62.	Chicken egg	Egg & products
63.	Duck egg	
64.	Quail egg	
65.	Turkey egg	
66.	Guinea fowl egg	
67.	Catfish	Fish & products
68.	Crab	
69.	Crayfish	
70.	Periwinkle	
71.	Other types of fish (titus, sower, tilapia etc)	
72.	Powdered milk	Milk and related products
73.	Evaporated milk	
74.	Yoghurt	
75.	Traditional (fura) milk	
76.	Soymilk	
77.	Butter/Bama	Fats and oils
78.	Vegetable/groundnut oil	
79.	Palm oil	
80.	Garlic	Condiments and spices
81.	Ginger	
82.	Locust beans	
83.	Onions	
84.	Dried pepper	
85.	Homemade fruit juice	Drinks
86.	Kunu drink	
87.	Sweetened drinks (coca cola etc)	
88.	Chips or pop corn	Desserts and snacks
89.	Cakes or muffins	
90.	Pastry & pies	
91.	Doughnut	
92.	Candy	

Table S2: Household Food Insecurity Access Scale (HFIAS)

No	Question	Response	Code
1	In the past four weeks, did you worry that your household would not have enough food?	a. Yes<br>b. No *(skip to Q2)*	
1a	How often did this happen?	a. Rarely<br>b. Sometimes<br>c. Often	
2	In the past four weeks, were you or any household member not able to eat the kinds of foods you preferred because of a lack of resources?	a. Yes<br>b. No *(skip to Q3)*	
2a	How often did this happen?	a. Rarely<br>b. Sometimes<br>c. Often	
3	In the past four weeks, did you or any household member have to eat a limited variety of foods due to a lack of resources?	a. Yes<br>b. No *(skip to Q4)*	
3a	How often did this happen?	a. Rarely<br>b. Sometimes<br>c. Often	
4	In the past four weeks, did you or any household member have to eat some foods that you really did not want to eat because of a lack of resources to obtain other types of food?	a. Yes<br>b. No *(skip to Q5)*	
4a	How often did this happen?	a. Rarely<br>b. Sometimes<br>c. Often	
5	In the past four weeks, did you or any household member have to eat a smaller meal than you felt you needed because there was not enough food?	a. Yes<br>b. No *(skip to Q6)*	
5a	How often did this happen?	a. Rarely<br>b. Sometimes<br>c. Often	
6	In the past four weeks, did you or any other household member have to eat fewer meals in a day because<br>there was not enough food?	a. Yes<br>b. No *(skip to Q7)*	
6a	How often did this happen?	a. Rarely<br>b. Sometimes<br>c. Often	
7	In the past four weeks, was there ever no food to eat of any kind in your household because of lack of<br>resources to get food?	a. Yes<br>b. No *(skip to Q8)*	
7a	How often did this happen?	a. Rarely<br>b. Sometimes<br>c. Often	
8	In the past four weeks, did you or any household member go to sleep at night hungry because there was not enough food?	a. Yes<br>b. No *(skip to Q9)*	
8a	How often did this happen?	a. Rarely<br>b. Sometimes<br>c. Often	
9	In the past four weeks, did you or any household member go a whole day and night without eating anything because there was not enough food?	a. Yes<br>b. No *(Finished)*	
9a	How often did this happen?	a. Rarely<br>b. Sometimes<br>c. Often	
